# JMJD6 orchestrates a transcriptional program in favor of endocrine resistance in ER+ breast cancer cells

**DOI:** 10.3389/fendo.2022.1028616

**Published:** 2022-11-07

**Authors:** Partha Das, Aritra Gupta, Kartiki V. Desai

**Affiliations:** National Institute of Biomedical Genomics, P.O.N.S.S, Kalyani, India

**Keywords:** estrogen receptor, RET, Tamoxifen, RNA-seq, MAPK pathway

## Abstract

High expression of Jumonji domain containing protein 6 (JMJD6) is strongly associated with poor prognosis in estrogen receptor positive (ER+) breast cancer. We overexpressed JMJD6 in MCF7 cells (JOE cells) and performed RNA-seq analysis. 76% of differentially expressed genes (DEGs) overlapped with ER target genes. Pathway analysis revealed that JMJD6 upregulated a larger subset of genes related to cell proliferation as compared to ER. Interestingly, JOE cells showed a decrease in ER target gene expression prompting us to check ER levels. Indeed, JOE cells showed a significant decrease in both ESR1 and ER levels and JMJD6 siRNA transfection increased the expression of both. Additionally, JOE cells showed increased RET and ERK1 expression, events associated with resistance to endocrine therapy. Accordingly, JOE cells displayed lower sensitivity and survived better at higher doses of 4-hydroxy tamoxifen (Tam) as compared to parental MCF-7 cells. Conversely, LTED-I and TAM R that resist Tam induced death, showed high expression of JMJD6. Further, JMJD6 siRNA treatment decreased growth and improved Tam sensitivity in TAM R. Comparison of JOE DEGs with known Tam signature genes showed a substantial overlap. Overall, these data suggest that blocking ER alone in patients may not eradicate proliferation of JMJD6 expressing ER+ cells and JMJD6 may predispose and sustain endocrine therapy resistance. We propose that immunostaining for JMJD6 could be developed as a potential marker for predicting endocrine therapy resistance. Further, antagonizing JMJD6 action in women expressing higher amounts of this protein, may offer a greater clinical benefit than endocrine therapy.

## Introduction

As per recent estimates by the National Cancer Institute, approximately 67 to 80% of breast tumors are positive for estrogen receptor (ER) expression, and they are dependent on its signaling for growth (GLOBOCAN, 2020). ER activity is blocked using either Selective Estrogen Receptor Modulators (SERMs) such as Tamoxifen to treat pre-menopausal women and Selective Estrogen Receptor Degraders (SERDs) like Fulvestrant/Faslodex are used in the metastatic setting (1, 2). Approximately 30% of women treated with tamoxifen (Tam) develop insensitivity to hormone therapies within 5-10 years, thereby limiting the success of this therapy (3–5). Though, various pathways to endocrine therapy resistance have been identified, very few markers for resistance have been established in the clinic (6).

Previously, we established that Jumonji domain-containing protein 6 (JMJD6) regulated cell cycle genes and that both EZH2 and JMJD6 regulate the E2F and DREAM target genes involved in this increased cell proliferation (7). In addition, high expression of JMJD6 was an indicator of unfavorable prognosis in ER+ breast cancer (8). JMJD6 is an epigenetic regulator of gene expression having lysyl hydroxylase and histone arginine demethylase activity. Its structure, function and activity in cancer initiation and progression is reviewed in detail by Yang et al. (9). JMJD6 has since become an important prognostic marker and a viable therapeutic target across multiple cancers. Recently, a small molecule inhibitor of JMJD6 (iJMJD6) has been tested in cell lines and patient derived xenograft systems and found to oppose cancer cell growth and progression of ER+ breast cancer (10).

JMJD6 also participates in ER regulated gene expression using both enzymatic activities (8, 11–13). Gao et al. showed that JMJD6 enhances hormone induced ER transcriptional activity by promoting RNA pol II phosphorylation and its release from paused or stalled sites (14). JMJD6 demethylates Arg residues in ER to promote its nuclear localization to regulate its non-genomic actions (13). Therefore, JMJD6 appears to positively regulate ER action in the presence of hormonal stimulus. However, despite Tam mediated blockade of ER, higher JMJD6 expression remains associated with poorer prognosis in women undergoing endocrine therapy (8). Therefore, the high JMJD6 levels may have a functional bearing on response to Tam. Our preliminary observations from Gene set enrichment analysis (GSEA) of microarray data in MCF7 cells overexpressing JMJD6 showed similarity with patterns found in Tam resistant cell lines and xenograft studies. JMJD6 appears to have functions beyond that of promoting ER target gene expression in ER+ breast cancer. In this paper, we have explored the transcriptional program elicited by JMJD6 in MCF7 cells and its contribution to endocrine therapy response. Our data suggests that high JMJD6 expression predetermines the eventual development of endocrine therapy resistance.

## Materials and methods

### Cell culture

MCF7 cells were purchased from the American Type Culture Collection (ATCC, VA, USA). The cells were cultured in Dulbecco’s modified Eagle’s medium (DMEM) (GIBCO, USA) with 5% fetal bovine serum (GIBCO, USA) and 1% Penstrep (GIBCO, USA) in humidified 5% CO_2_ incubator at 37°C. Cell lines were negative for mycoplasma presence (Lonza, Switzerland). Construction of wild-type JMJD6 tagged with V5 and generating stable clones of this construct in MCF7 cells (JOE) has been described earlier [denoted as JOE 1, JOE 2, and so on] (8). Cells transfected with empty vector (Vec) were used as a control. 17β-estradiol (E_2_) and 4-Hydroxytamoxifen (4-OHT) (Sigma Aldrich, USA) were used at concentrations indicated in the respective experiments.

### Transient transfection

MCF7 cells were transiently transfected for 24 hours (h) with V5 tagged JMJD6 using lipofectamine 2000 (Invitrogen, CA, USA) according to the manufacturer’s instructions. For siRNA, the reverse transfection protocol was carried out using JMJD6 siRNA (J6 siRNA; 5’-gcuauggugaacacccuaatt-3’) (8), ESR1 siRNA (Dharmacon: L-003401-00) and scrambled siRNA (ScsiRNA) was used as control (Ambion: 4635). Cells were harvested 48 h after transfection.

### Generation of TAM R and LTED cells

MCF7 cells were maintained in DMEM-F12 (GIBCO, USA) without phenol red, 10% Charcoal-dextran treated fetal bovine serum (CDFBS) (GIBCO, USA), 1% Penstrep and 1µM 4-OHT for 6 months (15). Tam resistance was confirmed by increasing 4-OHT up to 5 µM (data not shown). These cells are designated as TAM R. Long term estrogen deprived (LTED) cells were generated from MCF7 cells as described earlier (16).

### RNA-seq library preparation and data analysis

RNA was isolated from JOE and Vec cells by using RNAeasy Qiagen mini kit (Qiagen, Germany). Libraries were prepared using Illumina True-Seq stranded library kit. Library preparation was done at the National Genomics Core at NIBMG. Paired end sequencing (50 million reads each) was done by NOVASeq. FASTQC check was performed to remove spurious data. FastQ files were initially trimmed by CutAdapt to remove the adapter sequences and were aligned with the human genome (GrCh38.1) by Hisat2.0 algorithm. Aligned sequences were processed by Featurecounts package for obtaining RNA/transcript counts. EdgeR package was used to obtain differentially expressed genes (DEGs) using Galaxy (www.usegalaxy.org). Cluster 2.0 and Tree View programs were used for heatmap generation and visualization. Pathway enrichment analysis was done by Enrichr (https://maayanlab.cloud) and preranked analysis in GSEA (https://gsea-msigdb.github.io/gseapreranked-gpmodule/v6/index.html). Top 10 significant pathways were chosen for further comparisons. Sequencing Data has been uploaded in GEO omnibus (GSE211031). For comparison, microarray data of MCF7 cells treated with siRNA (ESR1 KD) was downloaded from GEO (GSE 27473) and analyzed in GEO2R to get DEGs. Genes with adjusted *p* value ≤ 0.05 and those that overlapped with DEGs from JOE cells were selected for further analysis in Enrichr. Figures and graphs were plotted using Bioconductor R, ggplot2 and ggvenn packages.

### Quantitative real time polymerase chain reaction

RNA from cell lines was isolated using Trizol (Ambion) as per the manufacturer’s instruction. One microgram of total RNA was reverse transcribed using SuperScript^®^ III First-Strand Synthesis System (Invitrogen, CA, USA). SYBR green mix (KAPA BIOSYSTEMS, South Africa) was used for quantitative real-time PCR (qRT-PCR), using primers described in **Supplementary Table 1**. C_T_ values of gene specific primers were normalized to C_T_ value of β-actin. Fold change in gene expression across samples was calculated by the formula $2^{\left(-\Delta \Delta C_{T}\right)}$ using values from ‘Vec’ cells converted to fold change 1.

### Western blot analysis

Cells were lysed in RIPA buffer containing 1X Protease Inhibitor Cocktail (Sigma Aldrich, USA), quantified by BCA method (Pierce, USA) and equal amounts (50 microgram) were analyzed on 10 – 12% SDS–PAGE gels. Proteins were transferred using PVDF membrane (Sigma Aldrich, USA). Primary antibodies were incubated at 4°C overnight and suitable HRP conjugated secondary antibodies for 1 hour at room temperature. Signals were detected using HI-ECL reagent (Biorad, USA) using Chemidoc (Biorad, CA, USA). Antibodies used were JMJD6 (PSR sc-28348, Santa Cruz Biotechnology; CA, USA; 1:1000), V5 for only exogenously expressed V5-tagged JMJD6 (R960-25, Invitrogen; 1:1000), ER-α (HC-20; F10) (Santa Cruz Biotechnology 1:1000), β Actin (A2228, Sigma Aldrich, USA;1:500), MAPK (ERK1/2) (137F5, Cell Signaling Technologies, 1:1000), Phospho-MAPK (ERK1/2) (Thr202/Tyr204) (197G2, Cell Signaling Technologies 1:1000), RET (3220, Cell Signaling Technologies 1:1000), Phospho-Akt (Ser473) (D9E4060 Cell Signaling Technologies 1:1000), Akt (Cell Signaling Technologies, 1:1000).

### Immunofluorescence microscopy

Cells were plated in chamber slides (Eppendorf, Germany) for 24 h. 10% Neutral buffered formalin (NBF) was used as a fixative. Cells were washed in 1X PBS followed by permeabilization (1X PBS+ 0.2% Tween-20), washed with Glycine (Sigma Aldrich, USA) and non-specific sites were blocked with 10% normal goat serum for 1 h. Cells were incubated with the primary antibody overnight (JMJD6 1:250, ER 1:250), washed and incubated with the appropriate secondary antibody (1:500) for 1 h. Signals were captured in a confocal microscope (Nikon Eclipse Ti2E). All experiments were performed in triplicates.

### Luciferase assays

Vector and JOE cells were cultured in E_2_ deprived media for 72 h before transfection. 10^5^ cells/well were plated in a 24 well plate, p(ERE)_2_ luciferase and pRL-TK plasmids were transfected using lipofectamine 2000 (Invitrogen, USA). Cells were treated with either ethanol (Veh), 10 nM E_2_, or 1 μM 4-OHT for 24 h and lysed in 1X Passive Lysis buffer (PLB). Reporter gene activity was measured using a Dual Luciferase Assay System (Promega, USA). ‘Veh’ samples were assigned the value of 1 and the remaining treatments were normalized to obtain fold change in luciferase activity.

### TCGA and KM plot analysis

For TCGA analysis, mRNA expression z-scores relative to all samples (log RNA-seq V2 RSEM) from ER+ Breast cancers from Breast Invasive Carcinoma (Cell, 2015) were used. Correlation plot was generated using www.cbioportal.org. Kaplan Meier plots were generated at www.kmplot.com/analysis using ER+ Breast cancer data. A ratio of JMJD6/ESR1 expression was used to divide the patients into two groups and their association with survival outcome was estimated.

### Cell proliferation and cell viability assay

Cell proliferation assay was performed with and without E_2_ treatment for both Vec and JOE cells. In brief, cells were starved in E_2_ depleted media for 3 days and plated at a density of 10^4^ cells per well (Day 0). 10 nM E_2_ was added after 24 h and media was replaced each day. Cells were counted in Hemocytometer at time points mentioned in the figure. Cell viability assay was performed by CCK8 kit (Sigma Aldrich, USA). Briefly, cells were starved for 3 days in E_2_ deprived media at a density of 10^4^ cells/well. Different doses of 4-OHT as indicated were added and cells were incubated for 72 h. The media was changed every day and absorbance was measured at 450 nm.

### Tamoxifen sensitivity assay in TAM R cells

TAM R cells were reverse transfected with JMJD6 siRNA as described above. ScsiRNA was used as a control. After 48 hours, a portion of the cells were subjected to western blot analysis to confirm downmodulation of JMJD6. Remaining TAM R cells were treated with Ethanol (Veh) or 1 µM 4-OHT (Tam). Cell numbers were counted in Hemocytometer at time points mentioned in **Figure 7D**.

### Statistical analysis

Each experiment was performed independently at least three times. Data are presented as mean ± standard deviation for each experiment except for RNA-Seq analysis. Statistical analysis was performed using GraphPad prism software. Comparisons between two groups were performed using student’s t-test and data was considered statistically significant at *p*≤ 0.05. Significance in all figures is indicated as follows: * *p* ≤ 0.05, **p ≤ 0.01, ***p ≤ 0.001 and ****p ≤ 0.0001.

## Results

### JMJD6 elucidates a transcriptional program that overlaps with ER mediated gene expression

**Figure 1:**
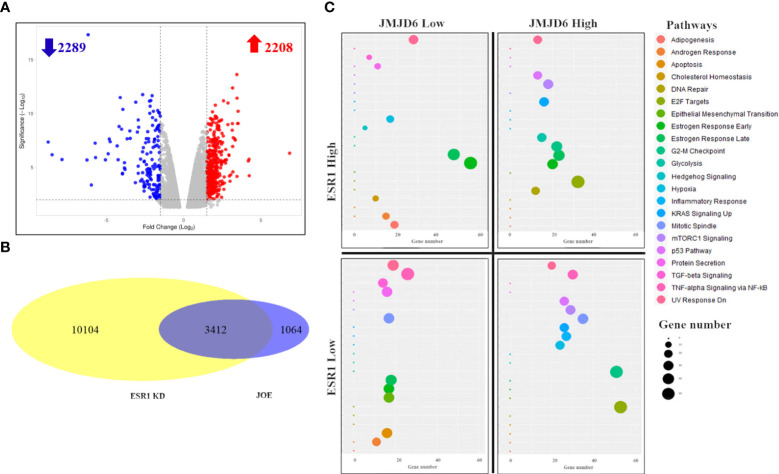
RNA-Seq analysis of JOE cells. **(A)** Volcano plot of differentially expressed genes. Red dots are upregulated genes and blue dots are downregulated genes. **(B)** Venn Diagram showing the number of overlapping genes between JOE and ESR1 KD cells. **(C)** Representative figure showing enrichment of pathways in 4 subsets of 3412 overlapping genes from the JOE and ESR1 KD sets. 4 quadrants show the enrichment of pathways per subset of genes. Induced by JMJD6 and ESR1 (top right), induced by JMJD6 but suppressed by ESR1 (bottom right); and so on. Each panel depicts a bubble plot with number of genes in each pathway on the X-axis versus the name of the pathway on Y-axis. The pathway and bubble size key are indicated on the right.

RNA-seq of JOE and Vec cells identified 4497 significantly differentially expressed genes (adjusted p value ≤ 0.05) following EdgeR analysis. This set of genes is denoted as the “JOE” set. A volcano plot of these genes is shown in **Figure 1A**. 2208 genes were upregulated and 2289 genes were downregulated by JMJD6. To find the highly regulated genes, a 2-fold change(2FC) cut off was imposed on the significantly changed DEGs and 1131 genes were obtained (**Supplementary Table 2**). We studied pathway enrichment using both GSEA preranked analysis and Enrichr methods and identified E2F signaling pathway, G2-M checkpoint, mTORC1 signaling, ESR1 mediated early and late gene expression, TGF-β signaling and others as significantly enriched pathways (**Supplementary Figure 1**). Of these, we have previously described contributions of JMJD6 to regulation of E2F target genes and the TGF-β signaling pathway (7, 8).

We explored estrogen early/late response pathways further and compared the JOE set genes with publicly available dataset for ER regulated genes (MCF7 cells treated with ESR1 siRNA, ESR1 KD set) (17). This dataset was chosen since the JOE dataset was obtained in the absence of E_2_ treatment. In the ESR1 KD dataset, 13516 DEGs were obtained (adjusted p value ≤ 0.05). Intersection of these two sets indicated that 3412, that is 76% of JOE set genes, overlapped with ESR1 KD set (**Figure 1B**). Though overlapping, the directionality of gene expression (induced vs repressed) was not conserved, and genes could be divided into 4 subsets based on the treatment and direction of change in gene expression. **Figure 1C** graphically represents the four quadrants and pathway enrichment analysis from each subset of genes. Details such as number of genes in each quadrant and the Enrichr output for top 10 pathways are represented as a heatmap and bar graph in **Supplementary Figure 2**. Both JMJD6 and ESR1 showed concordance in the regulation of the Estrogen early/late gene response pathway. Similarly, they both suppressed the TGFβ signaling, apoptosis and cholesterol biosynthesis pathways. Androgen Receptor (AR) response genes were suppressed by JMJD6, whereas, ER appeared to enhance this pathway. However, certain pathways showed distinct regulation in the JOE and ESR1 KD sets. JMJD6 induced genes (n=1634) showed enrichment for E2F, G2-M transition and mTORC1 signaling pathways (**Figure 1C**). The same set of genes was split into two groups in the ESR1 KD set; 742 genes were less expressed in presence of ER and 892 showed higher expression. For example, the G2-M transition genes were divided into two groups-repressed by ER and induced by ER. Further, while JMJD6 induced Mitotic spindle genes and TNFα signaling pathway, ER suppressed these genes. This suggests that JMJD6 probably induced a larger repertoire of cell proliferation genes as compared to ER (**Supplementary Table 3**). These data suggest that high JMJD6 may offer ER+ cells an additional advantage *via* executing an alternate pathway to cell proliferation and this pathway appears to be independent of the E_2_ -ER axis.

### Validation of JMJD6 and ER target genes

**Figure 2:**
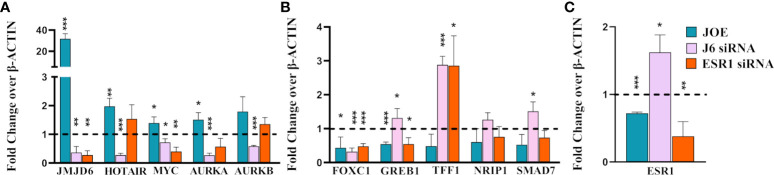
Realtime PCR analysis of representative genes in JOE, J6 siRNA and ESR1 siRNA treated MCF7 cells. **(A)** JMJD6 and JMJD6 regulated genes **(B)** ER regulated genes **(C)** ESR1. Vector and Scrambled siRNA treated samples were used as respective controls and values were normalized to 1 (shown as a dotted line). *p ≤ 0.05, **p ≤ 0.01, and ***p ≤ 0.001.

We validated candidate genes from DEG list that were a) previously known targets of JMJD6 (HOTAIR and MYC); b) genes that are known to be regulated by ER and JMJD6 upon E_2_ stimulation (TFF1, NRIP1, SMAD7, FOXC1, GREB1) and c) genes that have JMJD6 binding sites in their regulatory region (AURKA, AURKB) (7, 14, 18, 19). Data for all candidate genes in another JOE clone and western blots to represent successful down regulation of JMJD6 and ER post siRNA treatment is shown in Supplementary **Figure 3**. We found an increase in the expression of HOTAIR, MYC and AURKA in JOE cells which could be reversed by JMJD6 siRNA treatment (**Figure 2A**). qRT-PCR data obtained for ESR1 regulated genes was in accordance with microarray ESR1 KD data. ESR1 siRNA decreased the expression of MYC and AURKA. However, change in AURKB levels was not statistically significant. Interestingly FOXC1, GREB1, TFF1, NRIP1 and SMAD7 genes that are induced by and require binding of both JMJD6 and ER to their regulatory sites in the presence of E_2_, were repressed in JOE cells. Further, except for FOXC1, the expression of these genes was decreased in MCF7 cells following siRNA mediated knock down of JMJD6 (**Figure 2B**; **Supplementary Figure 3**). This suppression in ER target expression both in presence and absence of JMJD6 was curious, since previously published data suggested that JMJD6 was necessary for ER target gene expression in presence of E_2._ It was more likely that ER target genes would display higher expression in the presence of recombinantly expressed JMJD6 or they remain unaltered since JOE cells were not treated with E_2_ in our study (14). Unless 1) supraphysiological levels of JMJD6 negatively regulated ER transactivation function in the absence of hormone or 2) JMJD6 suppressed basal expression of ER target genes in absence of E_2_ or 3) high JMJD6 expression affected the levels of ER itself. We tested some of the possibilities and checked the expression levels of ESR1 mRNA in JOE cells. We found a decrease in ESR1 expression (**Figure 2C**), and in MCF7 cells transfected with JMJD6 siRNA ESR1 levels appeared to be higher than cells transfected with scrambled control (**Figure 2C**). These data suggested that JMJD6 may negatively affect the ER axis in breast cancer cells by depleting ER expression levels.

### JMJD6 depletes ER levels

**Figure 3:**
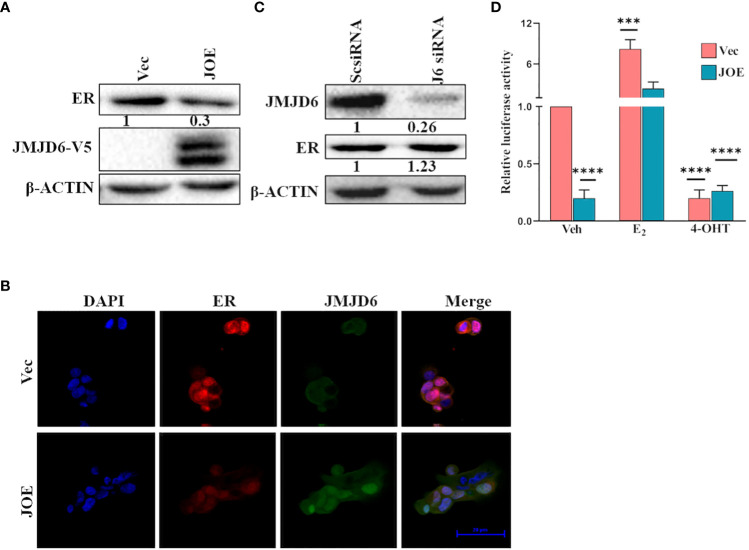
JMJD6 downregulates ER. **(A)** Immunoblot analysis of ER in JOE cells. β-Actin was used as internal control to demonstrate equal loading of proteins. Numbers below immunoblot panels represent densitometric scanning data plotted as a mean of three independent experiments. **(B)** Immunofluorescence staining of Vector and JOE cells using DAPI (blue), JMJD6 (green) and ER (red) and merged images (Scale bar, 20 μm). **(C)** Western blotting of ER protein after JMJD6 siRNA treatment of MCF7 cells (J6siRNA). Scrambled siRNA (ScsiRNA) was used as a control. Numbers below immunoblot panels represent densitometric scanning data plotted as a mean of three independent experiments. **(D)** ERE-Luciferase activity in JOE cells. The ratio of Firefly to Renilla luciferase activity in ethanol (Veh) treated Vec cells was normalized to 1 and fold change (FC) over Vec was calculated for all other samples. Data are representative of three independent experiments.

In JOE cells, immunoblot analysis indicated that expression of ER protein was lower than in Vec cells (**Figure 3A**). This observation was consistent in several clones stably expressing recombinant JMJD6 tagged with V5 (**Supplementary Figure 4A**). In addition, decrease in ER protein was evident after transient transfection of JMJD6 in MCF7 cells for a period of 24 h (**Supplementary Figure 4B**). Using immunofluorescence, we found that nuclear ER staining in JOE cells was lower as compared to Vec cells, and no ER specific fluorescence was detected in the cytoplasm (**Figure 3B**). Therefore, JMJD6 decreased ER but did not appear to affect its cellular localization. Knock down of JMJD6 by siRNA resulted in a slight but consistent increase in ER levels in MCF7 cells (**Figure 3C**).

Next, we tested ERE luciferase activity in JOE and Vec cells to determine if the residual ER in JOE cells could be activated by hormone stimulation and if high JMJD6 suppressed basal ER activity. As expected from the reduction in ER levels, the basal ERE-Luc activity was lower in JOE cells than Vec cells, however, it increased by at least 10-fold in the presence of E_2,_ and 4-OHT treatment negated this induced activity (**Figure 3D**). A similar 10-fold induction was evident in Vec cells following E_2_ treatment though the basal ERE-luc activity in these cells was far higher due to presence of substantial ER protein levels. We conclude that thought elevated levels of JMJD6 impinge upon total ER RNA/protein levels, decrease basal activity of ERE-luc, they appear not to interfere with the capacity of ER to transactivate gene expression in the presence of E_2._

### JMJD6 and ER expression is negatively correlated and high JMJD6:ER ratio associates with poorer survival outcome in ER+ patients

**Figure 4:**
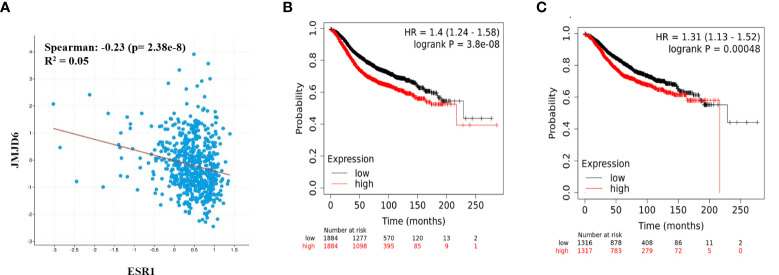
JMJD6 and ER in breast cancer patient samples. **(A)** Correlation between JMJD6 and ESR1 in ER+ breast cancer patient samples from TCGA data set. Kaplan-Meier survival curves plotted using the ratio of JMJD6/ESR1 RNA expression (n=3768) **(B)**, and ratio of JMJD6/ER proteins (n=2633) **(C)** in ER+ breast cancer patients.

Since JMJD6 appeared to suppress ER, we explored the clinical ramifications of this observation in publicly available datasets. In ER+ patient samples from TCGA breast cancer data, JMJD6 and ESR1 showed a trend towards negative correlation (Spearman, r^2^ = -0.23) (**Figure 4A**). We studied if this negative correlation had any bearing on patient outcome. We used the ratio of JMJD6 and ESR1 mRNA and JMJD6 and ER protein to test their association with survival. A high ratio would indicate high JMJD6, low ER samples and a low ratio would identify samples having low JMJD6, and hence higher level of ER. Interestingly, in more than 2500 ER+ samples, a high JMJD6 to ESR1 (RNA level) and a high JMJD6 to ER ratio (protein level) significantly associated with poorer prognosis and patient survival (**Figures 4B, C**).

### JMJD6 induces additional markers of Tam resistance

**Figure 5:**
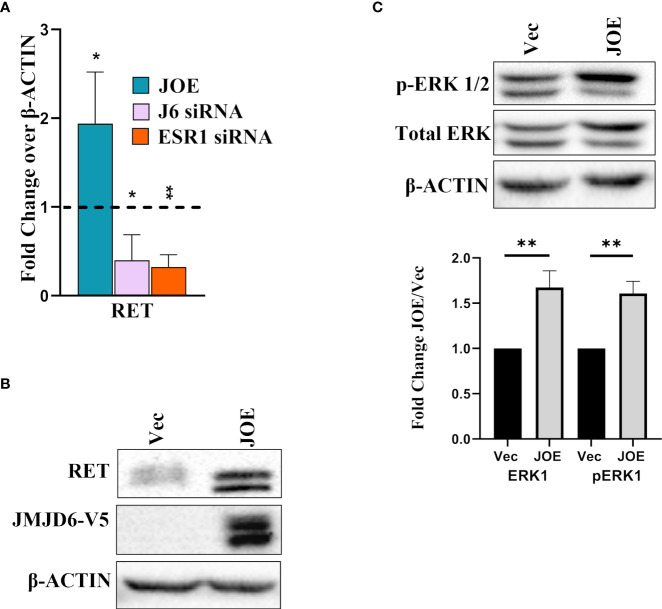
Effect of JMJD6 on known pathways in Tam insensitivity/resistance. **(A)** Western blot analysis of RET in Vec and JOE cells. **(B)** RET mRNA levels in JOE cells, J6 siRNA and ESR1 siRNA by qRT-PCR. Vector and Scrambled siRNA treated samples were used as respective controls and values were normalized to 1 (shown as a dotted line) **(C)** Western blot of total ERK 1/2 and p-ERK 1/2 in Vector and JOE cells. Lower Panel shows data obtained from densitometric scanning of ERK western blots. ERK expression levels in Vec cells were normalized to 1 and expression in JOE cells is shown as FC over 1.

Reduction in ER amounts is a well-known indicator of tamoxifen insensitivity (6, 20, 21). Other pathways involved in lower responsiveness include higher expression of RET (REarranged during Transfection), a receptor tyrosine kinase, and upregulation of the Mitogen Activated protein kinase (MAPK) pathway (22–25). In JOE cells, RET was induced at both RNA and protein level (**Figures 5A, B**). Knockdown of either JMJD6 or ESR1 in MCF7 cells using gene specific siRNAs suppressed the expression of RET (**Figure 5A**). This is not surprising since it is a well-known transcriptional target of ER (26). In addition, Tam resistant tumors display higher MAPK (ERK1/2) levels (27). Immunoblot analysis showed higher protein expression for both total ERK1 and p-ERK1 in JOE cells as compared to Vec cells (**Figure 5C**). Based on these data, we propose that JMJD6 may be involved in predisposing cells to Tam insensitivity by decreasing ER, increasing RET expression and total ERK1 levels in ER+ breast cancer cells.

### JMJD6 levels are higher in TAM R and LTED cells and JMJD6 regulates TAM R signature genes

**Figure 6:**
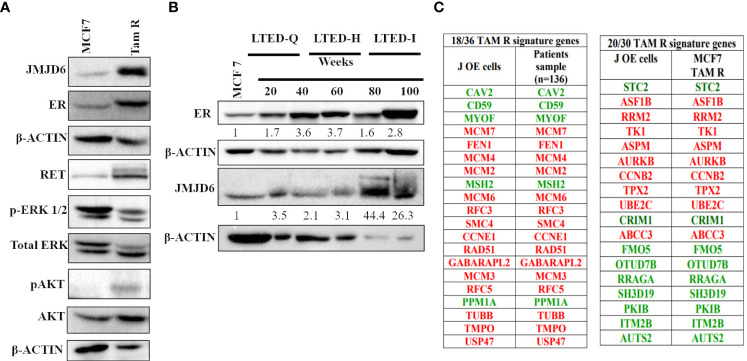
JMJD6 expression in MCF7 derived models of tamoxifen resistance (TAM R and LTED cells). **(A)** Characterization of TAM R cells **-**Western blot analysis of JMJD6, RET, ERK and AKT with β-Actin serving as an internal control to demonstrate equal loading of proteins. **(B)** JMJD6 protein expression determined by western blot of LTED cells at different weeks following E_2_ deprivation. **(C)** Overlap between DEGs in JOE cells and tamoxifen resistance signature genes obtained from published literature. Genes colored red are highly expressed and those colored green have lower expression in Tam resistant cells.

We studied the expression of JMJD6 in two models of Tamoxifen resistance that are derived from parental MCF7 cells; TAM R cells and LTED cells (28). LTED cells have different sensitivity to estrogen (LTED-Q- quiescent; LTED-H- hypersensitive; LTED-I- Independent) (29). Our premise was that if JMJD6 associated with Tam insensitivity, TAM R and LTED-I cells would show higher JMJD6 expression. Accordingly, TAM R cells displayed elevated levels of JMJD6 and pAKT but not ERK1/2, when compared to parental MCF7 cells (**Figure 6A**). LTED-Q and LTED-H cells did not possess high JMJD6 protein levels. Interestingly, the property of Tamoxifen’s agonist behavior in LTED-I cells, coincided with an increase in JMJD6 protein expression (**Figure 6B**).

Further, comparison of DEGs from JOE cells with published tamoxifen resistance gene signatures was undertaken. Analysis of gene expression data from 136 tamoxifen resistant patients had reported a signature of 36 genes (30). Of these, 18 genes appeared in the JOE dataset. Of note is the compete conservation of the direction of gene regulation (high expression vs low expression) in JOE cells and patient samples (**Figure 6C**). A second 30 gene signature has been derived from Tam R cells originating from MCF7, T47D and ZR75.1 (31, 32). Of these 30 genes, 20 genes were present in the JOE dataset with complete conservation of the direction of gene expression. These data clearly indicate that high JMJD6 levels may impose Tam insensitivity in ER+ breast cancer cells.

### JMJD6 lowers the sensitivity of JOE cells to Tam treatment but maintains responsiveness to E_2_

**Figure 7:**
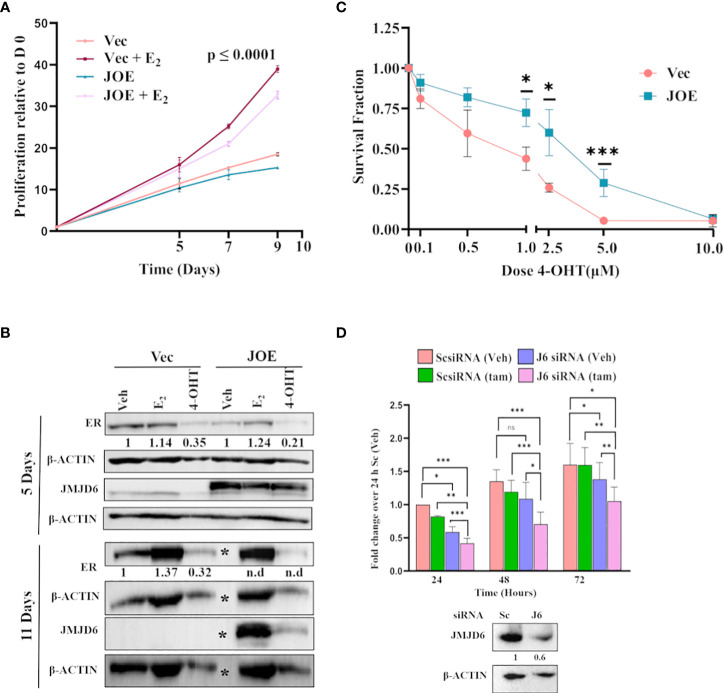
Effect of E_2_ and 4-OHT in JOE cells. **(A)** Cell proliferation assay in the presence of estrogen. Untreated Vec and JOE cells, and Vec cells treated with the hormone were used for comparison. **(B)** Western blot of E_2_ and Tam treated Vec and JOE cells after 5 and 11 days of E_2_ treatment. ‘*” denotes vehicle treated JOE cells that do not survive more than 10 days in E_2_ deprived media, therefore, no protein is available on day 11. Numbers below immunoblot panels represent densitometric scanning data plotted as a mean of three independent experiments (n.d.- not determined) in Vec cells, ER levels after E_2/_Tam were normalized to ER levels from ‘Veh’ treated Vec cells; In JOE cells, they were normalized to ER levels from ‘Veh’ treated JOE cells **(C)** Cell viability assay for MCF7 and JOE cells in the presence of different doses of 4-OHT. **(D)** JMJD6 siRNA and Tam treatment of TAM R cells. Upper panel: Data from ScsiRNA transfected cells treated with Veh for 24 h was normalized to 1 and data for J6siRNA and Tam treatment at different time points are expressed as fold change over 1. Lower panel: Immunoblot showing depletion of JMJD6 following siRNA treatment. Numbers indicate means of densitometric scanning analysis.

The ERE luciferase activity increased in JOE cells following E_2_ treatment indicating that the ability of lower levels of ER expression did not affect its ability to transactivate gene expression. Since ER induces cell proliferation in MCF7 cells, we assessed if E_2_ could do so in JOE cells. Further, we determined if E_2_ was capable of increasing ER levels to mediate this change. Our data reveal that E_2_ not only increased JOE cell proliferation but also recovered the expression of ER in these cells (**Figures 7A, B**). Since JOE cells were responsive to E_2,_ we studied the effect of 4-OHT on cell viability in Vec and JOE cells. At each dose of 4-OHT, JOE cells consistently showed a trend towards better survival than Vec cells, except at the highest dose of 10 μM Tam (**Figure 7C**). As shown, while only 30-45% of Vec cells survived at 1 and 2.5 μM doses, 60-75% of JOE cells remained alive. Therefore, JOE cells display lower sensitivity to Tam induced cytotoxicity

To check if JMJD6 directly contributed to Tam resistance, we treated TAM R cells with JMJD6 siRNA. 48 hours are plating, reduction in JMJD6 levels was confirmed by immunoblotting (**Figure 7D**). 1 μM Tam was added at this time and cell proliferation was monitored for the next 24, 48 and 72 h (**Figure 7D**). JMJD6 depletion led to a significant decrease in TAM R proliferation as compared to ScsiRNA treatment. More importantly, treatment of cells with both JMJD6 siRNA and Tam decreased cell proliferation further. Together these data raise the possibility that higher JMJD6 expression predisposes ER+ cancers to endocrine therapy resistance.

## Discussion

Our earlier paper indicated that high expression of JMJD6 in ER+ breast cancers was associated with poor survival despite treatment of these women with endocrine therapy (Tam) (8). Here we test if JMJD6 participates in altering sensitivity of MCF7 cells to Tam, and in MCF7 derived LTED and Tam R cells. By RNA seq and pathway analysis of JOE cells, we identified about 4500 genes that showed significantly altered gene expression as compared to the control Vec cells (**Figure 1A**). Interestingly, as shown in **Figure 1B**, 76% (3412 genes) of the JOE set were also regulated when MCF7 cells were studied in presence of ESR1 siRNA (ESR1 KD) (17). Because our RNA-seq data was obtained in the absence of hormone treatment, we compared the ESR1 KD profiles with JOE set genes instead of MCF7 datasets after E_2_ treatment. Gao et al. have previously shown that upon E_2_ treatment, JMJD6 and ER interact with each other to enhance ER target gene expression and JMJD6 siRNA could interfere with this upregulation (14). Interestingly, the extent of overlap indicated that high levels of JMJD6, in the absence of an activated ER pathway, also seemed to transcriptionally modulate a similar set of genes. Our data indicates a potential ‘degeneracy’ in regulation of the ER cassette genes and suggests that JMJD6 may hijack ER function when highly expressed. Our earlier immunohistochemical analysis showed high presence of JMJD6 in higher grade, stage III/IV and poorly differentiated ER+ tumors (8). Therefore, we explored the nature of pathways enriched in the 3412 genes. Overall, JMJD6 and ER induced and suppressed the same genes across the Estrogen early/late response, TGF-β signaling, cholesterol biosynthesis and Apoptosis pathways. Of note was the distinct regulation of E2F response, G2-M transition and the Mitotic spindle and the Androgen response genes in the JOE and ESR1 KD set. While all E2F and G2-M genes were upregulated by JMJD6, only a portion of them were induced and the rest were suppressed by ER (**Supplementary Table 3**). The E_2_-ER axis is thought to induce cell proliferation to promote cancer (33) and we have previously shown that JMJD6 also increases MCF7 proliferation and that it induces cell cycle and E2F regulated genes (7). JOE cells were not treated with E_2_ in our study but showed upregulation of many more cell proliferation genes. One of the mechanisms that JMJD6 may adopt to enhance proliferation gene expression could be *via* its ability to induce myc expression (34). These two proteins physically interact with each other, are known to engage with super-enhancer regions and both mediate promoter-proximal pause release at stalled RNA pol II sites to induce gene expression (35). Consequently, in ER+ tumors, high expression of JMJD6 may allow breast cancer cell proliferation by alternate means, in the absence of E_2_ or when blockade of the E_2_-ER axis occurs during endocrine therapy.

We chose 2 sets of genes to validate by qRT-PCR in JOE, Vec and JMJD6/ER siRNA treated MCF7 cells (**Figure 2**). First set consisted of genes that were known to be JMJD6 targets and they displayed an expected pattern of expression, that is, induced by JMJD6 and suppressed by JMJD6 siRNA (7). The second set comprised of ER target genes tested by Gao et al. (14). Here, while siRNA suppressed expression of ER target genes, high level of JMJD6 also suppressed their expression. Our qRT PCR assays were done in the absence of E_2,_ unlike the experiments in the previous paper and JMJD6 ChIP-seq data did not reveal JMJD6 binding sites in regulatory regions of these genes in the absence of E_2_ (data not shown) (14). Nevertheless, high JMJD6 negatively impacted the ER target gene expression in the absence of any hormone treatment. Since, these genes required the presence of both JMJD6 and ER, we wondered if high JMJD6 expression altered ESR1/ER levels in JOE cells. ESR1 levels were significantly depleted and subsequent analysis of ER protein by immunoblotting and immunofluorescence confirmed lower amounts of ER expression in JOE cells (**Figure 3**). This JMJD6 induced depletion of ER could explain the decrease in ER target gene expression. However, because in ERE-luc assays, E_2_ treatment increased luc activity, we cannot rule out the possibility that E_2_ may induce these genes in JOE cells. A similar suppression of ER and GREB1 is also observed when Enhancer of Zeste homolog 2 is highly expressed (36). We have shown that JMJD6 and EZH2 are almost always co-expressed in breast cancer cells and regulate E2F and DREAM target genes (7). Whether these two regulators also collaborate to suppress ER and thereby the expression of its target genes, needs to be explored further.

To check if our observations remain valid in human patient samples, we used publicly available TCGA and KM plot breast cancer datasets. Interestingly JMJD6 and ESR1 showed a trend towards negative correlation. Due to this observation, we compared patient groups based on a ratio of JMJD6 to ESR1 RNA/ER protein expression. Tumors expressing high JMJD6, but low ER levels were associated with poorer survival outcome when compared to those having higher expression of ER, that is lower ratio of JMJD6:ESR1/ER. Low ESR1 mRNA and low ER protein levels are significantly corelated with Tam and endocrine therapy resistance and patients suffering from these tumors appear to derive no clinical benefit from Tam treatment (20, 21). We imply that high JMJD6 levels could indicate the same. Taken together, these observations are now leading to the suggestion that JMJD6 may affect the response of cells to Tam/endocrine therapy.

Apart from ER levels, other indicators of Tam resistance are induction of RET (37) and increased levels of p-ERK1/2 (27). Interestingly, JOE cells displayed an increase in basal levels of RET and ERK1. However, RET is well known target of ER and unlike the other ER target genes, its expression was not diminished despite loss in ER levels in JOE cells. Recently, it was shown that ER, like JMJD6, also binds super-enhancers and ER absolutely requires Bromodomain containing protein 4 (BRD4) binding to regulate TFF1, GREB1, NRIP1 and other genes (38). RET was identified as a key downstream target of BRD4 and BRD4 siRNA decreased basal RET expression in MCF7 cells in the absence of E_2._ Curiously, JMJD6 partners with BRD4 and mediates transcriptional pause release. Its plausible that the loss of ER in JOE cells is compensated by the presence of BRD4, and a JMJD6-BRD4 interaction induces RET expression. RET leads to ligand independent activation of ER by phosphorylating Serine 118 (26, 39). However, since our data showed that JMJD6 led to loss in ER, this activity of RET may not be the primary mechanism of Tam insensitivity in tumors with high levels of JMJD6. Sunitinib, an inhibitor of RET, is often used to target the receptor tyrosine kinase in Tam resistance ER+ breast cancers (18, 30). Whether Sunitinib remains efficacious in tumors with high JMJD6 expression remains to be seen (40).

Gene signatures associated with Tam resistance have been documented in Tam resistant ER+ cell lines as well as in endocrine therapy resistant tumors. We checked if these signature genes appeared in our RNA seq data. 18/36 and 20/30 genes from two published TAM R signatures display the same TAM R pattern in JOE cells, strengthening our observation that JMJD6 may be associated with Tam insensitivity (**Figure 6C**). Overall, JMJD6 appears to initiate a transcriptional program leading to eventual Tam resistance in ER+ cells. If JMJD6 was responsible for regulation of TAM R genes, JMJD6 levels would be higher in cells resistant to Tam. We checked the expression of JMJD6 in TAM R and LTED cells that were generated in our lab. LTED-I cells grown in hormone deprived media for 80 and 100 weeks are known to be Tam insensitive and Tam acts as an agonist in these cells. We found high amounts of JMJD6 protein in LTED-I cells that were not present in the LTED-Q and LTED-H cells indicating that JMJD6 appeared as the cells became refractory to Tam’s antagonistic behavior (**Figure 6B**). TAM R cells showed a high expression of JMJD6, RET and increased level of pAKT, though levels of ERK remained uninduced (**Figure 6A**). Typically, high pERK levels TAM R cells occur after growth factor stimulation and these data are from untreated cells. On the other hand, increase in pAKT protein levels in resistant cells confer a survival advantage on these cells and they resist the cytotoxicity of high doses of Tam (41). In our TAM R cells, we also detected high levels of ER protein and different levels of ER protein have been reported earlier (22, 42–45). This is attributed to clonality of TAM R cells. It is plausible that JMJD6 uses RET/growth factor-based pathways to sustain Tam resistance in our cells.

To test the effect of Tam on Vec and JOE cells, we exposed them to varying concentrations of 4-OHT in culture (**Figure 7C**). While extremely high doses of 10 μM 4-OHT killed both cells, JOE cells consistently survived better at each dose of 4-OHT tested. Clearly, JMJD6 offered an advantage and cells were less sensitive to Tam treatment. To confirm the involvement of JMJD6 in Tam resistance, we transfected JMJD6 siRNA and assayed TAM R proliferation over 72 hours (**Figure 7D**). J6 siRNA alone decreased the proliferation of these cells over time. This is not surprising since involvement of JMJD6 in proliferation is clearly demonstrated earlier (8). In the presence of Tam, a further reduction of proliferation was observed. These data indicate that JMJD6 is not only essential for TAM R proliferation, but it’s loss sensitizes cells to Tam.

On the other hand, response of JOE cells to E_2_ remained intact. E_2_ induced proliferation of both Vec and JOE cells and we could show that it mildly recovered the expression of ER in JOE cells (**Figures 7A, B**). These data showed that the loss of ER in JOE cells was reversible. Moreover, the lowered ER levels were also capable of transactivating gene expression (ERE-luc) upon E_2_ treatment. High JMJD6 offered a curious dual advantage to the ER+ cells that could explain reasons of poorer survival of patients despite being treated with Tam. First, JMJD6 high cells appear to be less sensitive to Tam and second, they can respond to E_2_ by increasing their proliferation despite having lower ER levels to begin with. Collectively, based on all these observations, it is possible that increased expression of JMJD6 in breast cancer cells may result in lower response to endocrine treatment, particularly, Tam therapy.

## Conclusion

In the present study, we demonstrated that 76% of genes regulated by JMJD6 overlap with those altered by ESR1 siRNA treatment of MCF7 cells. Pathway analysis of these genes showed that JMJD6 conferred an additional ER independent axis of cell proliferation by promoting the expression of ER regulated proliferation genes in the absence of E_2._ JMJD6 decreased ER expression at both RNA and protein levels, and increased RET and ERK1 expression, pathways associated with resistance to Tam in patients. In addition several TAM R signature genes were found to be regulated by JMJD6. JMJD6 siRNA treatment alleviated ER suppression and decreased the expression of RET in MCF7 cells. Together, these data indicate that JMJD6 is an upstream regulator of multiple genes that are associated with Tam resistance. Next, we showed that higher expression of JMJD6 renders ER+ cells less sensitive to Tam, and conversely, in TAM R cells, its depletion recovers sensitivity to Tam. Therefore, high expression of JMJD6 maybe instrumental in lowering cellular response to Tam. We propose that JMJD6 levels may perform well as a valuable marker to determine response to endocrine therapy and the use of iJMJD6 as a viable therapeutic strategy to treat ER+ breast cancers involving high expression of JMJD6.

## Data availability statement

The data presented in the study are deposited in the GEO omnibus repository, accession number GSE211031.

## Author contributions

AG and KVD performed the RNA-seq analysis, comparison to other RNA-seq and publicly available data and made figures, AVG performed the immunofluorescence. PD developed the cell lines LTED and TAM R, performed siRNA experiments, hormone treatments and Western Blots. KVD conceived the project and the manuscript was written jointly by all 3 authors.

## Funding

This work was supported by Intramural Funding from National Institute of Biomedical. Genomics. PD is a graduate student supported by a CSIR fellowship (09/1033(0010)/2019-EMR-I). AG is a graduate student supported by DST Inspire fellowship (IF 180919).

## Acknowledgments

We thank Ms. Shayantani Chakraborty for technical assistance.

## Conflict of interest

The authors declare that the research was conducted in the absence of any commercial or financial relationships that could be construed as a potential conflict of interest.

## Publisher’s note

All claims expressed in this article are solely those of the authors and do not necessarily represent those of their affiliated organizations, or those of the publisher, the editors and the reviewers. Any product that may be evaluated in this article, or claim that may be made by its manufacturer, is not guaranteed or endorsed by the publisher.

## Supplementary material

Supplementary Figure 1
**(A)** EdgeR and **(B)** pre-ranked GSEA analysis: Prominent pathways regulated by JMJD6 in MCF7 cells

Supplementary Figure 2 Heatmap and pathway enrichment analysis of 3412 overlapping genes from the JOE and ESR1 KD set. Top ten pathways are represented in each subset of DEGs.

Supplementary Figure 3
**(A)** qRT-PCR analysis of candidate genes in additional JOE clone. Vec cell values were used as a control and normalized to 1 (shown as a dotted line). Depletion of **(B)** JMJD6 and **(C)** ER protein in MCF7 cells following gene specific siRNA treatment. Scrambled siRNA (ScsiRNA) is used as control. Numbers indicate means of densitometric scanning analysis.

Supplementary Figure 4
**(A)** Stable expression of JMJD6 tagged with V5 in MCF7 cells leads to decrease in ER levels in multiple selected clones. **(B)** Transient transfection of the same construct in MCF7 cells shows loss of ER protein expression. Numbers under the panel are representative of densitometric scans of 3 independent experiments. β-Actin is used as internal control for equal protein loading.

Supplementary Table 1 Primers used for qRT-PCR analysis.

Supplementary Table 2 JOE set genes (n=1131), Fold Change (FC) = 2, adjusted *p*-value < 0.05.

Supplementary Table 3 List of specific pathway genes and their level of expression in JOE and ESR1 KD datasets.
