# Supplementary figures and images for: JMJD6 orchestrates a transcriptional program in favor of endocrine resistance in ER+ breast cancer cells
Source: Front Endocrinol (Lausanne). 2022 Nov 7;13:1028616. doi: 10.3389/fendo.2022.1028616 (PMC9678079; doi:10.3389/fendo.2022.1028616)

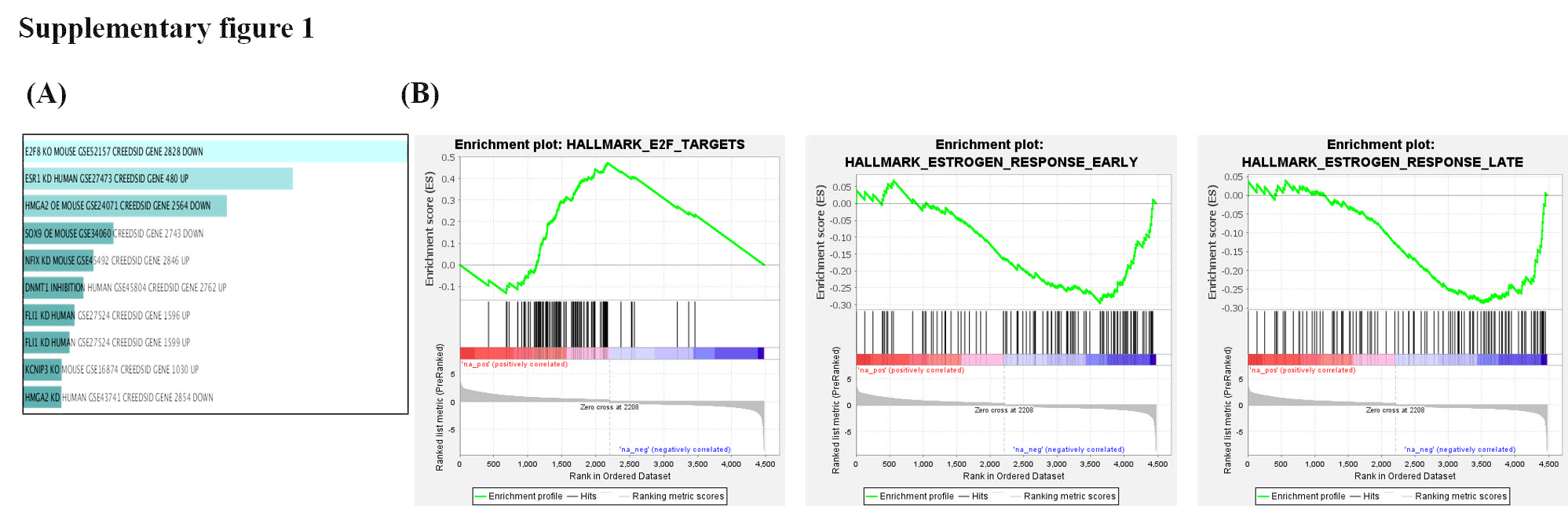

Supplement: Supplementary Figure 1 — (A) EdgeR and (B) pre-ranked GSEA analysis: Prominent pathways regulated by JMJD6 in MCF7 cells [file Image_1.tif]

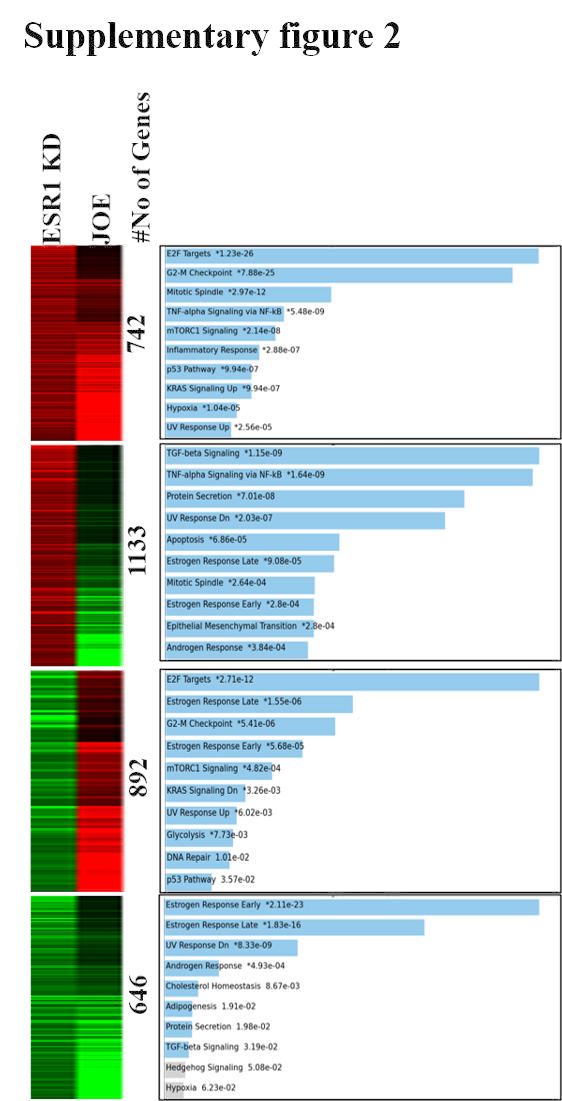

Supplement: Supplementary Figure 2 — Heatmap and pathway enrichment analysis of 3412 overlapping genes from the JOE and ESR1 KD set. Top ten pathways are represented in each subset of DEGs. [file Image_2.tif]

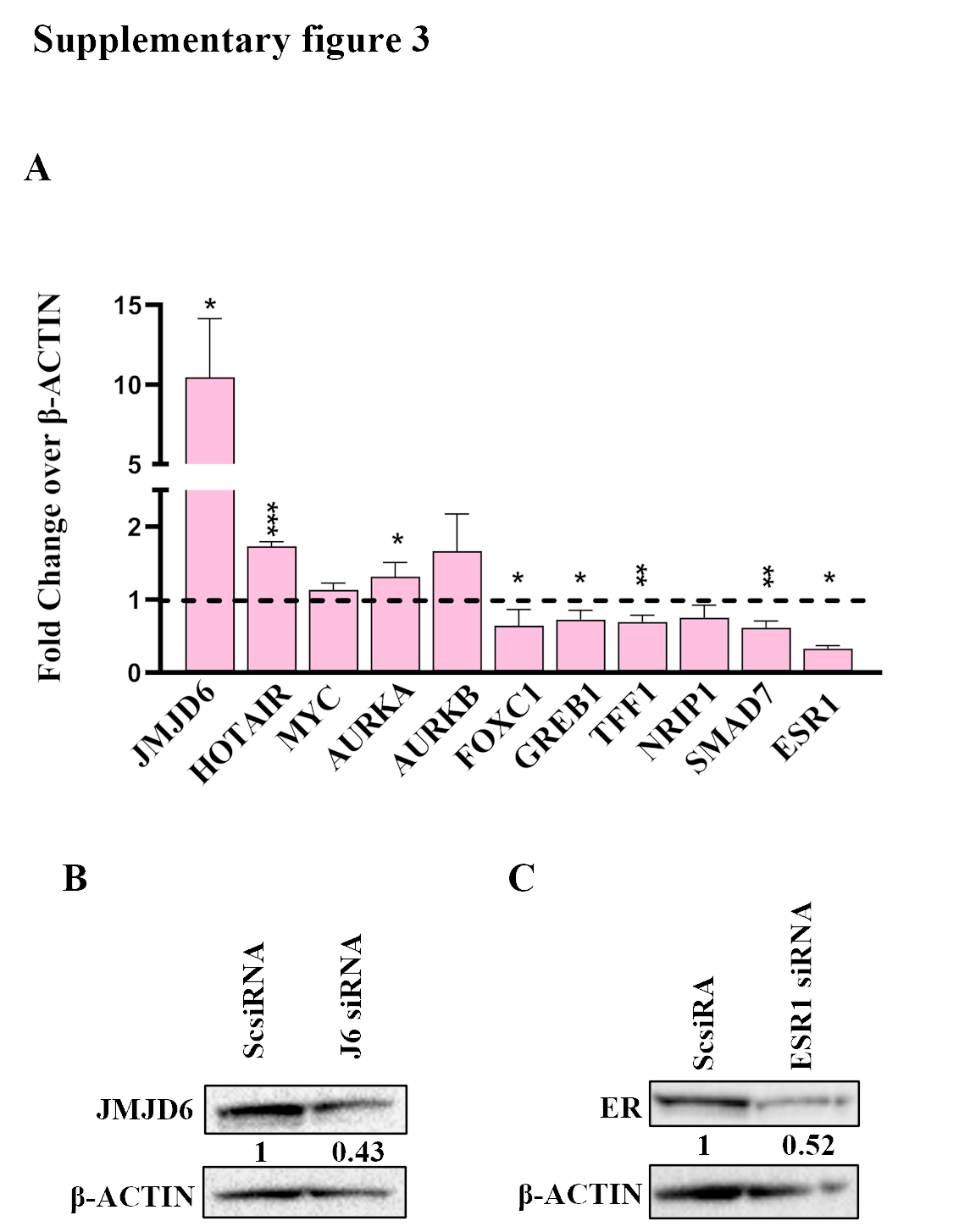

Supplement: Supplementary Figure 3 — (A) qRT-PCR analysis of candidate genes in additional JOE clone. Vec cell values were used as a control and normalized to 1 (shown as a dotted line). Depletion of (B) JMJD6 and (C) ER protein in MCF7 cells following gene specific siRNA treatment. Scrambled siRNA (ScsiRNA) is used as control. Numbers indicate means of densitometric scanning analysis. [file Image_3.tif]

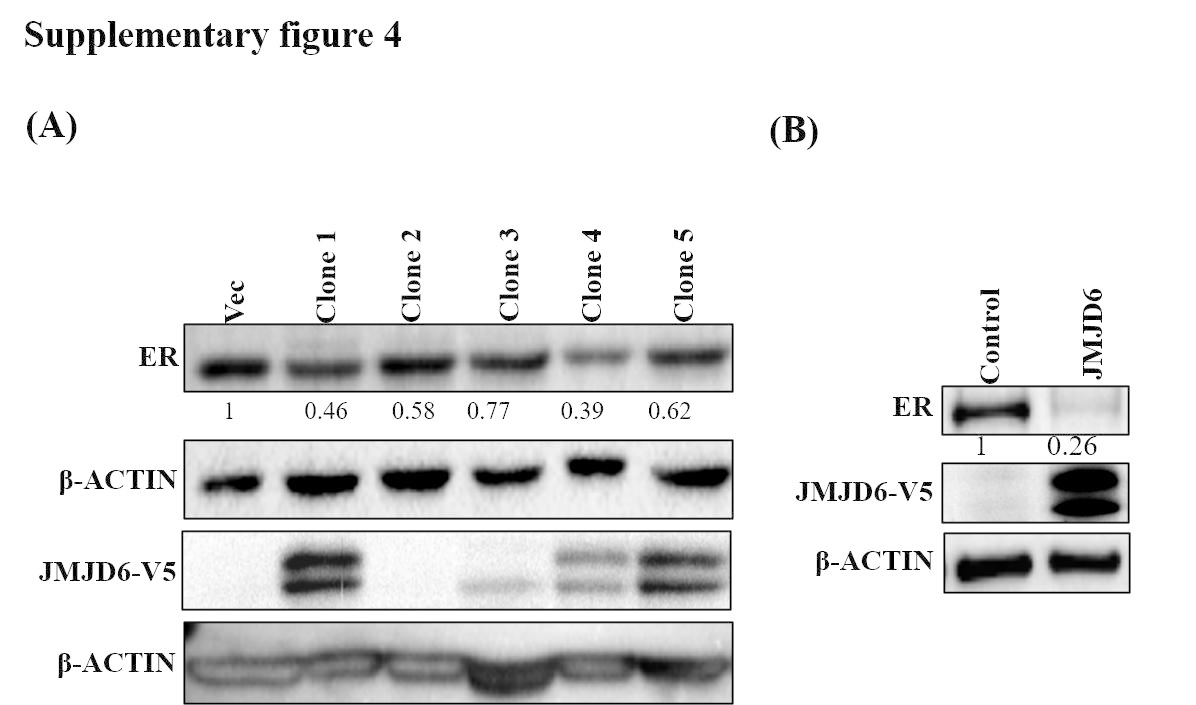

Supplement: Supplementary Figure 4 — (A) Stable expression of JMJD6 tagged with V5 in MCF7 cells leads to decrease in ER levels in multiple selected clones. (B) Transient transfection of the same construct in MCF7 cells shows loss of ER protein expression. Numbers under the panel are representative of densitometric scans of 3 independent experiments. β-Actin is used as internal control for equal protein loading. [file Image_4.tif]
